# Elevated thyroid stimulating hormone and metabolic syndrome risk in patients with first-episode and drug-naïve major depressive disorder: a large-scale cross-sectional study

**DOI:** 10.1186/s12888-024-05847-4

**Published:** 2024-05-21

**Authors:** Qiaoyang Zhang, Guanzhong Dong, Xuanyan Zhu, Yin Cao, Xiangyang Zhang

**Affiliations:** 1 Department of Psychology, The Affiliated Changzhou Second People’s Hospital of Nanjing Medical University, Changzhou, Jiangsu Province China; 2 Changzhou Medical Center, Nanjing Medical University, Nanjing, Jiangsu Province China; 3 Institute of Psychology, Chinese Academy of Sciences, 16 Lincui Road, Chaoyang District, Beijing, 100101 China; 4 Department of Psychology, University of Chinese Academy of Sciences, Beijing, China

**Keywords:** Metabolic syndrome, Depression, Thyroid, TSH

## Abstract

**Background:**

Metabolic syndrome (MetS) is common in major depressive disorder (MDD), but its relationship with thyroid hormones remains unclear. We aimed to examine the association of thyroid hormones and MetS in first-episode drug-naïve (FEDN) MDD patients.

**Methods:**

We recruited 1718 unmedicated MDD patients in this cross-sectional study. MetS was defined based on the 2004 Chinese Diabetes Society Criteria. Serum thyroid hormones including free thyroxine (FT4), free triiodothyronine (FT3), thyroid-stimulating hormone (TSH), thyroid peroxidase antibodies (TPOAb), and anti-thyroglobulin (TGAb) were examined. We used the logistic regression model to determine risk factors for MetS and examined the performance of the regression model by using the Area Under the Curve (AUC). In addition, we performed the trend test to test whether the results were robust.

**Results:**

The prevalence of MetS in unmedicated MDD patients was 34.4%. MDD patients with MetS had higher levels of serum TSH, TGAb, and TPOAb (all *P* < 0.001). Concurrently, serum TSH levels were independent risk factors for MetS in MDD patients (OR:1.49, 95%CI: 1.40–1.58), which could also distinguish MDD patients with and without MetS (AUC was 0.77). Additionally, in the trend test, the results also indicated a similar trend when TSH was used as a categorical variable (*P* for trend < 0.001).

**Conclusions:**

This study suggests that TSH levels were independent risk factors for MetS in FEDN MDD patients (OR:1.49). The examination of thyroid function may contribute to the early detection of MetS.

## Introduction

As the most debilitating disorders worldwide, metabolic syndrome (MetS) and major depressive disorder (MDD) are often reported as comorbid [1]. According to recent findings from the World Health Organization, MDD becomes the leading cause of disability globally, while people with depression in the world are estimated to be 4.4% [2]. MetS refers to a group of disorders including hypertension, insulin resistance, dyslipidemia, and obesity, which have the potential to lead to cardiovascular disease and type 2 diabetes [3]. MetS is steadily increasing and is estimated that will affect more than a billion people worldwide [4].

MDD patients were more likely to develop MetS, based on a meta-analysis, patients with MDD were 1.5 times more likely to have MetS when compared with controls in the general population [5, 6]. The high rates of MetS in MDD patients may be caused by unhealthy lifestyle habits, insulin resistance, central and peripheral inflammation, and hyperactivity of the hypothalamo-pituitary-adrenal axis [7–9]. Thyroid hormones are well known to participate in energy metabolism, and several studies conducted in the general population have reported a link between thyroid function and MetS [10–13]. Thus, a study from 2014 Korea National Health and Nutrition Examination Survey (*n* = 370) suggested that depressed individuals with subclinical hypothyroidism were more likely to meet the criteria for MetS [14]. However, the association of thyroid hormones and MetS in Chinese MDD patients has been little explored.

Furthermore, although the relationship between antidepressants and MetS remains controversial, it is widely believed that antipsychotics could contribute to MetS [15–19]. Therefore, to exclude confounding effects of medication, disease course, and comorbidities, we selected unmedicated MDD patients to examine the relationship between MetS and thyroid hormones. In this study, we aimed to: (1) compare serum thyroid hormones between MDD patients with and without MetS; and (2) identify the independent association of thyroid function with MetS risk. We hypothesized that there would be significant differences in thyroid function between MDD patients with and without MetS, with thyroid hormones being related to the increased risk of MetS in MDD patients.

## Methods

### Participants

All participants provided informed consent. The Institutional Review Board of the First Affiliated Hospital of Shanxi Medical University approved the study protocol (No.2016-Y27). After a comprehensive clinical evaluation, we recruited 1718 unmedicated MDD patients during 2015–2017. Inclusion criteria included (1) aged 16–65 years; (2) meeting MDD criteria according to the fouth edition of the Diagnostic and Statistical Manual of Mental Disorders (DSM-IV); (3) the overall score of the 17 item-Hamilton Rating Scale for Depression (HAMD-17) ≥ 24; (4) first-onset and no previous treatment. Exclusion criteria included (1) with a substance use disorder except for nicotine; (2) with any serious physical illnesses; and (3) being pregnant or breastfeeding.

### Demographics and clinical interview

Demographics and clinical variables were collected. Any self-injurious behavior that could lead to suicide was classified as a suicide attempt. To assess clinical symptoms of mood, the HAMD-17 and the Hamilton Anxiety Scale (HAMA) were used. A cut-off of 24 was used in the HAMD-17 examination to select participants [20]. Furthermore, psychotic symptoms were assessed using the Positive and Negative Syndrome Scale (PANSS) positive subscale. Patients scoring more than 15 were considered to have psychotic symptoms [21].

### Blood samples

Serum samples were obtained from each participant between 6 and 8 a.m after an overnight fast, and promptly delivered to the hospital’s laboratory center for analysis before 11 a.m on the same day. Lipid profiles including total cholesterol (TC), triglycerides (TG), high-density lipoprotein cholesterol (HDL-C), and low-density lipoprotein cholesterol (LDL-C) were detected by the enzymatic colorimetric assays. The fasting blood glucose (FBG) were measured by a Cobas E610 (Roche, Basel, Switzerland). The levels of free thyroxine (FT4), free triiodothyronine (FT3), thyroid-stimulating hormone (TSH), thyroid peroxidase antibodies (TPOAb), and anti-thyroglobulin (TGAb) were assessed using the Roche C6000 Electrochemiluminescence Immunoassay Analyzer (Roche Diagnostics, Indianapolis, IN, USA). As reported in our previous studies, we classified TSH into three groups: <2.5, 2.5–4.2, and ≥ 4.2 mIU/L [22].

### Metabolic syndrome

Blood pressure, height, and weight were assessed by trained nurses. Body mass index (BMI) was calculated using the formula: BMI = Weight (kg)/Height (m)^2^. MetS was defined according to the 2004 Chinese Diabetes Society Criteria [23, 24]. The criteria are that at least 3 of the following cardiometabolic risk factors are met: (1) BMI ≥ 25 kg/m^2^; (2) serum HDL-C < 0.9 mmol/L in men, or < 1.0 mmol/L in women; (3) TG ≥ 1.7 mmol/L; (4) systolic blood pressure (SBP) ≥ 140 mmHg, or diastolic blood pressure (DBP) ≥ 90 mmHg; and (5) FBG ≥ 6.1 mmol/L.

### Statistical analysis

Demographic characteristics of different groups of MetS were analyzed using chi-square test, independent samples t-test, or Mann-Whitney U-test. Bonferroni correction was adopted to adjust multiple tests. We then applied univariate analysis to identify risk factors for MetS in MDD patients. Factors with significant differences were entered into logistic regression (Forward: LR). In order to distinguish MDD patients with and without MetS, we evaluated the discriminative power of TSH using the the area under the receiver operating characteristic curve (AUC-ROC) method. In addition, the independent association between TSH and MetS in MDD patients was examined using multivariate logistic regression. Age, gender, marital status, and education were adjusted in Model 1. And model 2 was adjusted for disease duration, suicide attempts, psychotic symptoms, HAMD score, and HAMA score. To validate the results were robust and to examine the possibility of nonlinearity, we converted TSH into a categorical variable and conducted the trend test. *P* < 0.05 (two-sided) was considered significant. All analyses were performed with R 4.2.0.

## Results

### Comparison of MDD patients with or without comorbid MetS

Table 1 shows the proportion of MetS in MDD patients was 34.4% (591/1718). Compared to MDD patients without MetS, those who with MetS were more likely to be older, female, less educated, and married. In addition, MetS group had more suicide attempts, psychotic symptoms, and much higher HAMA, HAMD scores than non-MetS group (all *P* < 0.001). Serum TSH, TGAb, and TPOAb levels were higher in MDD patients with MetS when compared with those without MetS (all *P* < 0.001). Nevertheless, no differences were found in FT3 and FT4 levels within the two groups.

**Table 1:** Socio-demographics and clinical characteristics of subjects

Variable	Overall	MDD patients without MetS	MDD patients with MetS	*P*
N	1,718	1,127	591	
Age	34.0 (22.0)	32.0 (21.0)	39.0 (22.0)	**< 0.001** ^**b**^
Gender, n (%)				**< 0.001** ^**a**^
Male	588 (34.2)	432 (38.3)	156 (26.4)	
Female	1,130 (65.8)	695 (61.7)	435 (73.6)	
Education, n (%)				**< 0.001** ^**a**^
Less than high school	413 (24.0)	234 (20.8)	179 (30.3)	
High school	760 (44.2)	510 (45.3)	250 (42.3)	
College	449 (26.1)	321 (28.5)	128 (21.7)	
Higher than college	96 (5.6)	62 (5.5)	34 (5.8)	
Marital status, n (%)				**< 0.001** ^**a**^
Not married	502 (29.2)	362 (32.1)	140 (23.7)	
Married	1,216 (70.8)	765 (67.9)	451 (76.3)	
Illness duration, months	5.0 (3.0, 8.0)	4.5 (3.0, 8.0)	6.0 (3.0, 9.0)	**< 0.001** ^**b**^
Suicide attempts, n (%)				**< 0.001** ^**a**^
No	1,372 (79.9)	956 (84.8)	416 (70.4)	
Yes	346 (20.1)	171 (15.2)	175 (29.6)	
Psychotic symptoms, n (%)				**< 0.001** ^**a**^
No	1,547 (90.0)	1051 (93.3)	496 (83.9)	
Yes	171 (10.0)	76 (6.7)	95 (16.1)	
HAMD score	30.0 (28.0, 32.0)	30.0 (28.0, 32.0)	32.0 (29.0, 33.0)	**< 0.001** ^**b**^
HAMA score	21.0 (18.0, 23.0)	20.0 (18.0, 22.0)	21.0 (19.0, 24.0)	**< 0.001** ^**b**^
BMI, kg/m^2^	24.4 ± 1.9	24.0 ± 1.9	25.1 ± 1.8	**< 0.001** ^**c**^
SBP, mmHg	119.5 ± 10.9	116.5 ± 10.0	125.2 ± 10.3	**< 0.001** ^**c**^
DBP, mmHg	75.9 ± 6.7	74.4 ± 6.0	78.9 ± 7.1	**< 0.001** ^**c**^
TC, mmol/L	5.2 ± 1.1	5.0 ± 1.0	5.7 ± 1.1	**< 0.001** ^**c**^
TG, mmol/L	2.2 ± 1.0	2.0 ± 0.9	2.6 ± 0.9	**< 0.001** ^**c**^
HDL-C, mmol/L	1.2 ± 0.3	1.3 ± 0.3	1.0 ± 0.2	**< 0.001** ^**c**^
LDL-C, mmol/L	3.0 ± 0.9	2.8 ± 0.8	3.2 ± 0.9	**< 0.001** ^**c**^
FBG, mmol/L	5.4 ± 0.6	5.2 ± 0.6	5.8 ± 0.6	**< 0.001** ^**c**^
TSH, mIU/L	5.1 ± 2.6	4.3 ± 2.3	6.6 ± 2.4	**< 0.001** ^**c**^
FT3, pmol/L	4.9 ± 0.7	4.9 ± 0.7	4.9 ± 0.7	0.19 ^**c**^
FT4, pmol/L	16.7 ± 3.1	16.7 ± 3.2	16.7 ± 2.9	0.622 ^**c**^
TgAb, IU/ml	21.5 (14.4, 43.6)	20.7 (13.9, 33.8)	22.5 (15.6, 83.2)	**< 0.001** ^**b**^
TPOAb, IU/ml	17.4 (12.3, 34.6)	16.6 (12.2, 29.7)	19.9 (12.6, 54.5)	**< 0.001** ^**b**^

Bold indicated that the results were statistically significant

^a^Comparison was tested by χ^2^-test

^b^Comparison was tested by the Mann-Whitney U test

^c^Comparison was tested by independent - sample t-test

Abbreviations: MDD = major depressive disorder; MetS = metabolic syndrome; HAMD = Hamilton depression rating scale; HAMA = Hamilton anxiety rating scale; BMI = Body mass index; SBP = Systolic blood pressure; DBP = Diastolic blood pressure ; TC = Total cholesterol; TG = Triglycerides; HDL-C = High-density lipoprotein cholesterol; LDL-C = Low-density lipoprotein cholesterol; FBG = Fasting blood glucose; TSH = Thyroid Stimulating Hormone; FT3 = free triiodothyronine; FT4 = free thyroxine; TgAb = anti-thyroglobulin antibodies; TPOAb = thyroid peroxidases antibodies

### Independent risk factors for MetS in MDD patients

Factors with significant differences in univariate analysis were entered into the multivariable logistic regression for MetS. We included eleven variables (age, gender, marital status, illness duration, suicide attempts, psychotic symptoms, HAMA, HAMD, TSH, TgAb, and TPOAb) in the regression model. After controlling for confounding factors, the independent risk factors for MetS in MDD patients were as follows (Table 2): age (B = 0.02, *P* = 0.006, OR = 1.02), gender (B = 0.56, *P* < 0.001, OR = 1.75), HAMD score (B = 0.07, *P* = 0.006, OR = 1.08), and TSH (B = 0.40, *P* < 0.001, OR = 1.49).

**Table 2:** Factors associated with metabolic syndrome in MDD patients

Variable	B	Statistic	*P*	OR	95%CI
Age	0.02	2.76	0.006	1.02	1.01–1.03
Gender	0.56	4.46	< 0.001	1.75	1.37–2.25
HAMD score	0.07	2.73	0.006	1.08	1.02–1.13
TSH	0.40	13.26	< 0.001	1.49	1.40–1.58

Abbreviations: HAMD = Hamilton depression rating scale; TSH = Thyroid Stimulating Hormone

### Association of serum TSH and MetS risk in MDD patients

**Fig. 1:**
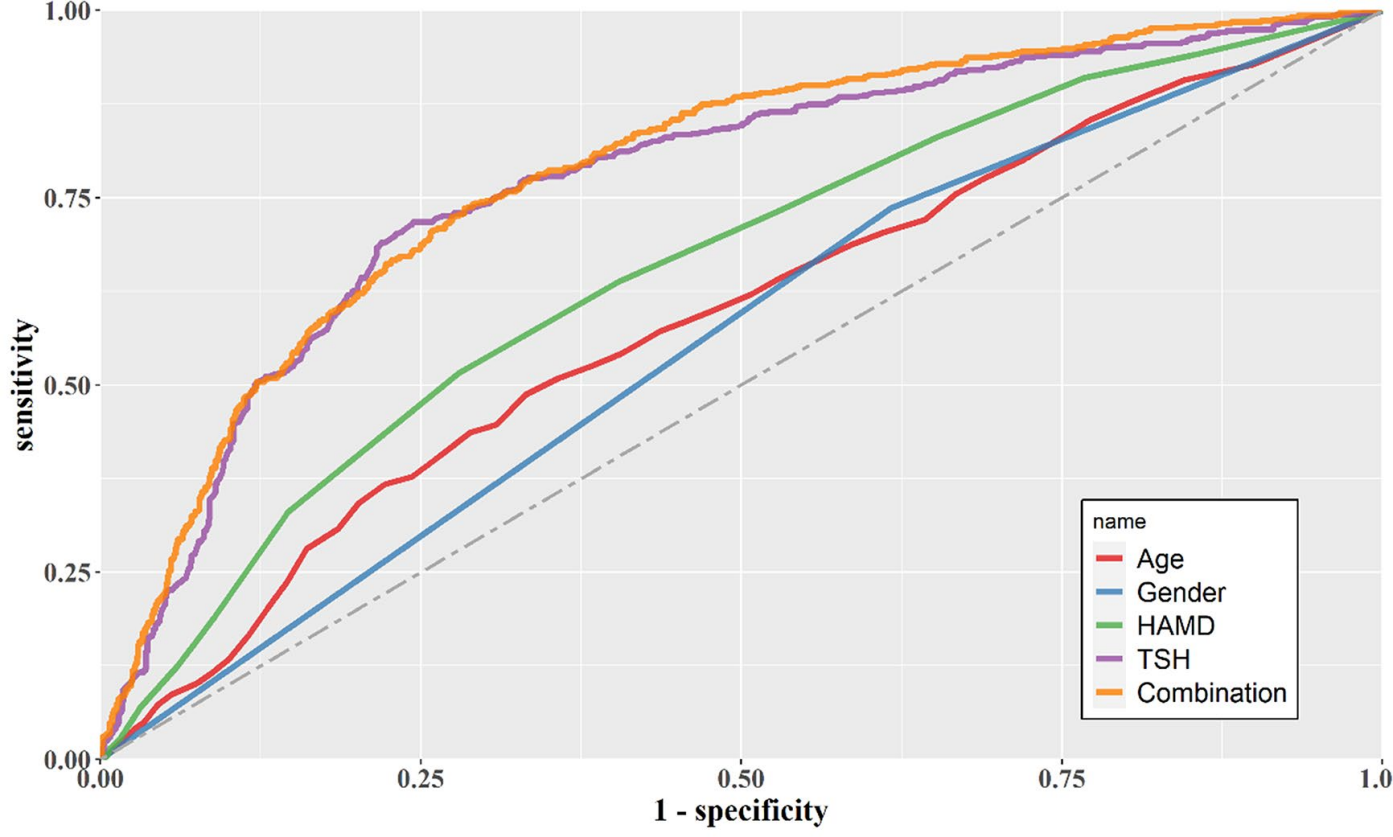
The discriminatory capacity of related factors for distinguishing between patients with and without MetS in MDD patients. The area under the curve of Age, Gender, HAMD score, and TSH were 0.59, 0.56, 0.66, and 0.77, respectively

The results of AUC ROC showed that TSH had the highest AUC value of 0.77, which could distinguish MDD patients with MetS from those without MetS. Furthermore, when we combined age, gender, HAMD score, and TSH, we found an AUC value of 0.78 which was close to the AUC value of TSH itself (Fig. 1).

In the trend test (Table 3), we stratified TSH into three subgroups (< 2.5, 2.5–4.2, and ≥ 4.2, respectively) and used the lowest subgroup (< 2.5) as the reference group. The results showed a similar trend when TSH was used as a categorical variable, with the highest TSH subgroup (≥ 4.2) having a higher risk of MetS than the lowest subgroup (< 2.5) (*P* < 0.001 for trend).

**Table 3:** Multivariable logistic regression analysis between TSH and MetS in MDD patients

TSH (categories)	No. patients	No. MetS (%)	Crude model	Model 1	Model 2
			OR (95%CI)	OR (95%CI)	OR (95%CI)
<2.5 mIU/L	290	32 (11)	Reference	Reference	Reference
2.5 to<4.2 mIU/L	381	61 (16)	1.54 (0.97–2.43)	1.52 (0.95–2.41)	1.42 (0.89–2.27)
≥ 4.2 mIU/L	1047	498 (47.6)	7.31 (4.97–10.77) *	7.35 (4.97–10.86) *	5.86 (3.89–8.84) *
*P* for trend			<0.001	<0.001	<0.001

Model 1 was adjusted for age, gender, education, and marital status

Model 2 was adjusted for age, gender, education, marital status, illness duration, suicide attempts, psychotic symptoms, HAMD score, and HAMA score

* *P* < 0.001

## Discussion

The present study found the relationship between thyroid hormones and MetS risk in MDD patients. The main findings were: (1) MDD patients with MetS had higher levels of serum TSH, TPOAb, and TgAb than those who without MetS; (2) Age, gender, TSH levels, and HAMD score were independent risk factors for MetS in MDD patients; and (3) TSH levels had an ability to discriminate between MDD patients with MetS and without MetS (AUC was 0.77).

We found a prevalence of 34.4% for MetS in our MDD patients, which was close to a previous meta-analysis that included 18 studies of depression defined by interview (*n* = 5,531) and reported a rate of 30.5% [6]. This study also found that older age was related to MetS risk in MDD patients, which is consistent with data from the general population and patients with severe mental illness [25–27]. One possible reason for this is the slowing of energy metabolism with age, along with the onset of many diseases, such as obesity, diabetes, and hypertension, which would contribute to a high MetS risk [28]. Furthermore, patients who had higher HAMD scores were at a significantly increased risk of MetS, which may be due to overlapping pathophysiology, socioeconomic factors, and lifestyle [6]. There is clear evidence that most patients with MDD adopt an unhealthy lifestyle with changes in diet and exercise, which increases the incidence of hyperglycemia and hypertriglyceridemia and therefore may further increase the MetS risk. Concurrently, according to this study, women were at a higher risk of developing MetS than men. MetS is believed to increase more rapidly with age in women than in men, mainly due to hormonal changes during and after menopause [29].

Importantly, we found that TSH levels in MDD patients were independently associated with increased MetS risk, but there was no relationship between MetS risk and FT3, FT4, TgAb, and TPOAb. For more than a decade, investigators have extensively investigated thyroid hormones and MetS, but the results have been inconsistent [30–32]. Recently, according to a Chinese Mendelian randomization study using 2,903 individuals with normal thyroid function, FT3 and TSH levels are positively related to MetS incidence [11]. The present study strengthens the evidence that TSH levels are associated with the MetS risk. Moreover, in a large Korean study (*n* = 4,775), TPOAb positivity was associated with MetS in euthyroid subjects (OR:1.39, 95% CI:1.05–1.84), suggesting thyroid autoimmunity plays a role in MetS [12]. However, we did not find an association between TPOAb and MetS risk in MDD patients. Several factors may contribute to the conflicting results described above, including different subjects, ethnicity, design, setting, the definition of MetS, and population iodine intake.

Thyroid hormones play a comprehensive role in each of components of MetS, engaging with diverse pathways and mechanisms. Thyroid dysfunction has a clear influence on body weight, primarily due to edema, and thyroid hormones can also contribute to obesity and dyslipidemia by modulating metabolic rates, lipid synthesis, and degradation [33]. In addition, thyroid dysfunction may lead to insulin resistance, thereby resulting in elevated blood sugar levels [34].

The present study has several important clinical implications. Firstly, our study indicate the need for a more comprehensive consideration of metabolic health in depression treatment. Incorporating thyroid function testing into the treatment plans of patients with depression may lead to better clinical outcomes. Secondly, preventive measures may be necessary in patients with depression to reduce the occurrence and progression of metabolic syndrome. This may include regular monitoring of thyroid function, lifestyle modifications, and adjustments to medication therapies. Thirdly, healthcare providers can better assess the overall health status of patients and develop more personalized treatment plans.

There are some limitations to this study that should be acknowledged. First, causality could not be inferred due to the cross-sectional design. Second, in addition to thyroid dysfunction’s impact on metabolic syndrome components, potential confounding factors like iodine intake, physical activity, smoking, and alcohol consumption may influence our study’s outcomes. Their complex interactions with metabolic pathways necessitate cautious interpretation. Third, a potential limitation is that there are no data on patients taking any antihypertensive, antidiabetic, or lipid-lowering drugs that may affect peripheral biomarkers.

## Conclusions

Our study showed that serum TSH levels were independent associated with MetS risk in first episode and drug naïve patients MDD patients. Thyroid function tests may be benefit to the prevention and early detection of MetS in MDD. Besides, more longitudinal studies are needed in the future to clarify the cause-and-effect relationship.

## Data Availability

The datasets used and analysed during the current study are available from the corresponding author on reasonable request.

## Abbreviations

AUC: Area Under the Curve
AUC-ROC: area under the receiver operating characteristic curve
DSM-IV: Diagnostic and Statistical Manual of Mental Disorders
FT4: free thyroxine
FT3: free triiodothyronine
FEDN: first-episode drug-naïve major depressive disorder patients
FBG: fasting blood glucose
HAMD: Hamilton Rating Scale for Depression
HAMA: Hamilton Anxiety Scale
HDL: High-Density Lipoprotein
MetS: metabolic syndrome
MDD: major depressive disorder
PANSS: Positive and Negative Syndrome Scale
TSH: thyroid-stimulating hormone
TPOAb: thyroid peroxidase antibodies
TGAb: anti-thyroglobulin
